# Theranostic Potential of a New ^64^Cu-Labeled NOTA-R954 Peptide Conjugate for Kinin B1R Expressing Prostate Cancer

**DOI:** 10.3390/pharmaceutics17091215

**Published:** 2025-09-18

**Authors:** Sadaf Ghanaatgar Kasbi, Martin Savard, Frédéric Couture, Céléna Dubuc, Véronique Dumulon-Perreault, Marie-Edith Nepveu-Traversy, Samia Ait-Mohand, Robert Sabbagh, Sameh Geha, Brigitte Guérin, Yves Dory, Fernand Gobeil

**Affiliations:** 1 Department of Pharmacology and Physiology, Faculty of Medicine and Health Sciences, Université de Sherbrooke, Sherbrooke, QC J1H 5N4, Canada; sadaf.ghanaatgar-kasbi@usherbrooke.ca (S.G.K.); martin.savard@usherbrooke.ca (M.S.); celena.dubuc@usherbrooke.ca (C.D.); marie-edith.nepveu-traversy@usherbrooke.ca (M.-E.N.-T.); 2 Institute of Pharmacology, Faculty of Medicine and Health Sciences, Université de Sherbrooke, Sherbrooke, QC J1H 5N4, Canada; brigitte.guerin2@usherbrooke.ca; 3 TransBIOTech, Lévis, QC G6V 6Z3, Canada; frederic.couture@tbt.qc.ca; 4 Department of Medical Imaging and Radiation Sciences, Faculty of Medicine and Health Sciences, Université de Sherbrooke, Sherbrooke, QC J1H 5N4, Canada; veronique.dumulon-perreault@usherbrooke.ca (V.D.-P.); samia.ait-mohand@usherbrooke.ca (S.A.-M.); 5 Department of Surgery, Division of Urology, Université de Sherbrooke, Sherbrooke, QC J1H 5N4, Canada; robert.sabbagh@usherbrooke.ca; 6 Department of Pathology, Centre Hospitalier Universitaire de Sherbrooke, Sherbrooke, QC J1H 5N4, Canada; sameh.geha@usherbrooke.ca; 7 Department of Chemistry, Faculty of Sciences, Université de Sherbrooke, Sherbrooke, QC J1K 2R1, Canada; yves.dory@usherbrooke.ca

**Keywords:** kinins, GPCR B1 receptor, peptide antagonist, theranostics, prostate cancer

## Abstract

**Background/Objectives**: This study explores the potential of the inducible G protein-coupled kinin B1 receptor (B1R) as a target for the diagnosis and treatment of prostate cancer (PCa) and aims to develop the first theranostic agent targeting hB1R for both molecular imaging and radionuclide therapy. **Methods**: B1R expression was analyzed via qPCR and immunohistochemistry in human PCa cells and tissues specimens. A novel ^64^Cu/NOTA-conjugated peptide analog of the potent B1R antagonist R954 was synthetized and evaluated in vitro and in vivo. **Results**: B1R was confirmed to be expressed (RNA, protein) by varying degrees in all PCa cell lines and tissues investigated, with protein level significantly correlating with tumor grades. This finding was supported by similar analyses from the TCGA and MSKCC databases. In vitro, the ^64^Cu/NOTA-βAla-R954 conjugate showed nanomolar affinity/potency at hB1R, complete plasma stability over 24 h, significant cellular uptake (up to 33% of ID at 24 h), and dose-dependent anti-clonal growth effects. In vivo, the radioconjugate remained stable in circulation for up to 90 min and was primarily excreted intact via the kidneys following IV administration. Intravenous ^64^Cu/NOTA-βAla-R954 (7.5 MBq) effectively detected subcutaneous PCa xenografts via µPET imaging in male athymic nude mice. At a single higher dose (65 MBq; 50 µg/kg), it significantly reduced tumor growth without observable toxicity. This antitumor effect was associated with increased apoptosis (active caspase-3) and reduced proliferation (Ki67), as shown by immunohistochemistry. In contrast, the nonradioactive ^Nat^Cu/NOTA-βAla-R954 had no therapeutic effect at the same dose. **Conclusions**: Our findings provide proof-of-concept for the potential theranostic use of ^64^Cu/NOTA-R954 in PCa, and potentially other types of B1R-positive solid cancers.

## 1. Introduction

Prostate cancer (PCa) is the most common non-skin cancer and the second leading cause of cancer deaths among men in North America [1]. In Canada, approximately 1 in 8 men will be diagnosed with PCa during their lifetime, and 1 in 30 cases will result in death [2]. Despite advances in diagnosis and treatment, mortality rates remain high for locally advanced (stage III) and metastatic (stage IV) cases, highlighting the need for improved therapies, early detection, and precise staging. Evidence indicates that timely and accurate diagnosis significantly improves prognosis, with early intervention before metastasis markedly increasing the 5-year survival rate [1]. However, PCa is one of the few cancers not well detected by conventional ^18^F-fluorodeoxyglucose positron emission tomography (PET) imaging, due to its low metabolic activity [3]. Thus, continuous innovation in diagnostic tools and therapeutic strategies remains essential to reducing the burden of advanced disease.

Currently, PCa screening relies on serum prostate-specific antigen (PSA) measurements and digital rectal examination (DRE), both of which have limited sensitivity and specificity [4]. When clinical suspicion persists, magnetic resonance imaging (MRI) and targeted biopsies may improve diagnostic accuracy. Nevertheless, developing reliable, non-invasive detection methods remains a critical challenge [5].

In this regard, a promising non-invasive approach to enhancing PCa screening across various stages is the use of targeted molecular imaging and radiotherapy through theranostics. These innovative agents combine diagnostic and therapeutic capabilities within a single molecule. Unlike traditional non-selective chemotherapy, theranostics enable precise identification of patients whose tumors express specific molecular targets, facilitating personalized treatment and predicting a more favorable response to radiation therapy [6,7].

Peptide-activated G protein-coupled receptors (GPCRs) are attractive biomarker targets for developing cancer theranostics due to their overexpression on cancer cell surfaces and their role in regulating key cellular processes critical for cancer, such as proliferation, apoptosis, migration, chemoresistance, angiogenesis, immune invasion, and metastasis [8,9,10,11]. However, to date, only a limited set of GPCR-targeted peptidic theranostics (e.g., analogs of somatostatin, vasointestinal peptide, and bombesin/gastrin-releasing peptides) have been developed for detecting and treating PCa, and their clinical applicability is currently under investigation [12,13,14,15,16,17]. In this context, the kinin B1 receptor (B1R) emerges as a highly promising target for theranostic applications. B1R overexpression is common in many human solid cancers, including PCa, contributing to the growth, migration, and invasion of cancer cells; see comprehensive reviews [18,19]. Studies have shown that exogenous stimulation of B1R by kinin desArg^9^-metabolites (linear peptides) increases the proliferation and migration of both androgen-sensitive and -insensitive human PCa cells in vitro [18,19]. Moreover, peptide B1R antagonists have been effective in inhibiting PCa cell growth both in vitro and in vivo in tumor-bearing nude mice [20]. Notably, B1R has minimal roles in normal physiology [21] and is induced by tumor-associated inflammation, reducing potential side effects. A pilot clinical study on a limited number of PCa biopsy specimens (16 cases) suggested a correlation between B1R expression and the onset of PCa [22]. B1R may, thus, be a prime candidate for PCa detection and targeted radiotherapy, using techniques like PET and novel biostable radiolabeled kinin B1R peptide analogs.

The main objectives of the study were to further investigate the clinical importance of B1R as a biomarker and therapeutic target for PCa using molecular profiling of relevant in vitro human PCa cell lines and clinical samples, and to evaluate the theranostic potential of a new radiopeptide targeting B1R for PCa detection and therapy. For this purpose, we employed our optimized lead peptide B1R antagonist, AcOrn[Oic^2^,^(αMe)^Phe5,dβNal^7^,Ile^8^]desArg^9^-bradykinin (codenamed R954), which was identified through structure–activity relationship studies and exhibits excellent potency, selectivity, specificity, and stability [23,24,25,26]. Among medical radionuclides, we selected the isotope ^64^Cu due to its growing recognition for theranostic applications in recent years, attributed to its unique decay properties that are well-suited for both PET imaging (t_1/2_= 12.7 h; β^+^, 0.65 MeV [17.8%]) and endoradiotherapy (β^−^, 0.58 MeV [38.4%], Auger electrons [43.5%]) [27,28,29,30]. In addition, our cyclotron facility, equipped with a PET imaging center, supplies this radionuclide on a biweekly basis at a high apparent molar activity (333–444 TBq/mmol). The selected in vitro and in vivo models were based on two human B1R-expressing PCa cell lines: LNCap and PC3 cells; these cell lines represent androgen-dependent and castration-resistant forms of PCa, respectively, and are widely used in preclinical studies to model hormone-sensitive and advanced, treatment-resistant diseases.

In this study, we present new evidence regarding the clinical utility of B1R as a biomarker for PCa therapy. We also demonstrate the efficacy of the newly designed B1R radioantagonist ^64^Cu/NOTA-βAla-R954 in targeting and limiting the growth of experimental PCa, highlighting its potential for future research in cancer theranostics.

## 2. Materials and Methods

### 2.1. Antibodies

Antibodies and their respective sources (in parentheses) are listed as follows: anti-active (cleaved) caspase-3 antibody (Cell Signaling Technology; 5A1, Danvers, MA, USA); anti-Ki67 antibody (Abcam; ab15580, Waltham, MA, USA); anti-CD34 antibody (Abcam; ab81289); anti-LYVE1 antibody (Abcam; ab14917); anti-nuclear pore complex antibody (Abcam; ab24609); anti-Na+/K+ ATPase α antibody (Santa Cruz Biotechnology; SC-28800, Dallas, TX, USA); anti-clathrin HC antibody (Santa Cruz Biotechnology; SC-58714); and anti-lamin A/C antibodies (Santa Cruz Biotechnology; SC-56139); anti-cytochrome C antibody (BD Biosciences; 556433, San Jose, CA, USA); anti-tubulin antibody (Sigma-Aldrich; T9026, Burlington, MA, USA); anti-LAMP-2 antibody (DSHB University of Iowa; clone H4B4, Iowa City, IA, USA); anti-calnexin antibody (Abcam; ab2798); rabbit anti-human B1R polyclonal (C-terminus) antibody LS-A799 (LifeSpan Biosciences Inc., Seattle, WA, USA); rabbit anti-human B1R antiserum AS434 targeting 6 distinct epitopes on the extra- and intracellular domains of B1R (donated by Dr W Muller-Esterl) [31,32]; non-immunized rabbit immunoglobulin fraction (Agilent Technologies, Santa Clara, CA, USA); goat anti-mouse and anti-rabbit HRP-conjugated secondary antibodies (Sigma-Aldrich and AbD-Serotec, Raleigh, NC, USA, respectively); and Alexa-488 conjugated anti-rabbit antibody (Molecular Probe, Life Technology, Eugene, OR, USA). Target specificity of the two anti-B1R antibodies LS-A799 and AS434 has been reported elsewhere [31,32,33] and has been further established herein by demonstrating relatively higher staining intensities obtained with these two primary antibodies in human embryonic kidney 293 cells (HEK293) stably transfected with human B1R in comparison with mock-transfected cells, as assessed by epifluorescence microscopy (see Supplementary Figure S1).

### 2.2. Tissue Microarray (TMA) Construction and Immunohistochemical (IHC) Analysis

Formalin-fixed paraffin-embedded (FFPE) archived prostate specimens from patients who underwent radical prostatectomy between 2006 and 2011 at the Centre Hospitalier Universitaire de Sherbrooke were used. Patients consented to participate and signed a consent form. The research protocol was approved by the Institutional Review Committee for the Use of Human Resected Material at the Centre Hospitalier Universitaire de Sherbrooke (approval #10-017). This study was conducted in accordance with the Declaration of Helsinki and approved by the Human Ethics Committee at the Centre de Recherche du CHUS (Protocol #2013-490; 24 October 2012), Sherbrooke. Consent to use paraffin-embedded prostate tissue specimens for the current study was obtained through telephone interviews from all subjects or their families, with appropriate written documentation.

For each prostate tissue, duplicate cores (1.5 mm diameter of both tumor primary pattern and secondary pattern as well as prostatic intraepithelial neoplasia (PIN) and normal prostate glandular tissues) were extracted from donor blocks and inserted into a new paraffin block. The nature of each core was confirmed by hematoxylin–eosin (H&E) staining and examined by an expert uropathologist (SG). One case of metastatic PCa obtained by fine needle aspiration of peripancreatic ganglia was also included in the study.

TMA sections (4 µm) were deparaffinized and stained with the anti-B1R antibody LS-A799 (1:400) using a Dako streptavidin–biotin-based automated stainer (Agilent Technologies, Santa Clara, CA, USA), and counterstained with hematoxylin. Stained sections were scanned in brightfield using the Hamamatsu Nanozoomer 2.0-RS whole slide scanner (Hamamatsu Corporation, Bridgewater, NJ, USA) and images exported with Nanozoomer Digital Pathology (NDP) view 2 imaging software (version 2.6.13; Hamamatsu Corporation. Images were then analyzed by 2 independent observers (FC and SG) using a pre-established scale for staining evaluation (see Supplementary Figure S2). Each core was scored individually and the highest scores from duplicates by each observer were used to calculate the average immuno-score.

### 2.3. Fresh Tissue Dissection and RNA Isolation

Prostate tissues used for RNA extraction were freshly (typically within 30 min) dissected from prostate specimens obtained from radical prostatectomies performed at the Centre Hospitalier Universitaire de Sherbrooke. The tissues were frozen at −20 °C with OCT compound (Tissue-Tek; Miles Scientific, Elkhart, IN, USA). Sections of 5 µm were cut and fixed in formalin for H&E staining to enable pathological examination. Tumor regions were delineated along with adjacent non-cancerous tissues by a clinical pathologist, and dissection was performed accordingly. The dissected tissues were washed with nano-pure RNase-free water (Wisent Inc., Saint-Jean-Baptiste, QC, Canada) to remove any visible traces of the OCT compound. The tissues were then finely ground in liquid nitrogen, and RNA was extracted using QIAGEN RNeasy spin columns (QIAGEN, Toronto, ON, Canada) according to the manufacturer’s instructions. RNA integrity (200 ng total) was assessed by agarose gel electrophoresis.

### 2.4. Expression and Survival from Databases

Expression levels of BDKRB1 and survival data were retrieved using cBioportal in the datasets: TCGA Prostate Adenocarcinoma (Previously known as TCGA Provisional) and MSKCC Cancer Cell 2010 [34]. For TCGA RNA-Seq data, a Z-score compared to diploid cells of 1.75 was used to distinguish high (altered) from low expressor (unaltered). For MSKCC microarray data, a z-score from mRNA expression values of 2.25 was used.

### 2.5. Cell Cultures

The PCa cell lines LNCaP (AR+/PSA+/p53wt), PC3 (AR-/PSA-/p53null), CWR22Rv1 (AR+/PSA+/p53mutated), and DU145 (AR-/PSA-/p53mutated) were purchased from the American Type Culture Collection (ATCC, Manassas, VA, USA). PCa cells were cultured in RPMI-1640 supplemented with 10% fetal bovine serum (FBS, Wisent Inc.) + 1% penicillin/streptomycin (Pen/Strep) and were maintained in a humidified 5% CO_2_ environment at 37 °C. The cells were passaged and collected using 0.05% trypsin/EDTA (Wisent Inc.) or StemPro Accutase^®^ solution (Life Technologies, Carlsbad, CA, USA). PCa cell lines were routinely screened for mycoplasma contamination using PCR-based detection methods. Initially, regular testing was performed with the Universal Mycoplasma Detection Kit from ATCC to confirm the absence of contamination. Subsequently, the cell lines were periodically verified at the RNomic platform of Université de Sherbrooke using the IVM Biotech Mycoplasma PCR Detection Kit (Cat. No. MDK001; IVM Biotech, Longueil, QC, Canada). Stable puromycin-resistant human embryonic kidney cells (QBI-HEK293A) overexpressing human B1R or B2R, achieved through lentiviral-mediated transduction, were cultured in Dulbecco’s modified Eagle’s medium (DMEM) supplemented with 10% of FBS and 1% Pen/Strep and passaged, as indicated above.

### 2.6. RNA Extraction and Quantitative Real-Time PCR Analysis

Total RNAs from human PCa cell lines and PCa tissues were isolated using TRIzol reagent (Invitrogen, Carlsbad, CA, USA) according to the manufacturer’s instructions. Genomic DNA was removed from total RNA using RNase-Free DNase (Qiagen). RNA integrity was assessed with a TapeStation System (Agilent Technologies). Then, 0.5 to 2 µg of total RNA was subjected to reverse transcription using Oligo-dT primers and Superscript III transcriptase (Invitrogen). Quantitative real-time PCR (qRT-PCR) was conducted on a MX3005p Stratagene instrument (Agilent technologies) or on a CFX OPUS-384 system (Bio-Rad, Hercules, CA, USA) with Brilliant III SYBR green (Agilent technologies). Primers used were B1R sense, 5′-CTTTTGGGAGGACTTCATTGAC-3′; antisense, 5′-GGGATGAAGATATTGGAGCAAG-3′; β-actin sense, 5′-GTTGCTATCCAGGCTGTGCTA- 3′; antisense, 5′-GCGGATGTCCACGTCACACTT-3′. Relative mRNA expression was analyzed with the comparative Ct method (2^−∆∆CT^) and normalized to β-Actin as an internal reference gene.

### 2.7. Gene Expression Analysis of CDX Models Using Affymetrix^®^ HG-U133 Plus 2.0 Arrays

B1R gene expression analysis of the human PCa cell line-derived xenografts (four CDX models) in Nu/Nu mice was conducted by Charles River custom services (Freiburg, Germany) [35]. For mRNA preparation, tumors were grown in untreated mice until they reached a size of 400–800 mm^2^. Following sacrifice, tumors were immediately excised, and tumor pieces free of necrosis were flash-frozen in liquid nitrogen. Following mechanical tissue disruption, total tumor RNA was extracted using the RNeasy Mini kit (QIAGEN). Gene expression profiles were obtained by Affymetrix HGU133plus2 microarray chips (Thermo Fisher Scientific, Waltham, MA, USA). Expression values were extracted from CEL files and calculated and log 2 transformed using R/Bioconductor packages (gcRMA).

### 2.8. Production of 64Cu

Copper-64 (^64^Cu) was prepared on a TR-19 or a TR-24 cyclotron (ACSI) via the ^64^Ni(p,n)^64^Cu reaction, using an enriched ^64^Ni target electroplated on a rhodium disc. The [^64^Cu]CuCl_2_ was recovered from the target following the procedure of McCarthy et al. [36] and subsequently converted to [^64^Cu]Cu(OAc)_2_ by dissolving the [^64^Cu]CuCl_2_ in ammonium acetate (0.1 M; pH 5.5).

### 2.9. Peptide Synthesis and NOTA Conjugation

The peptide R954 was synthesized on a Symphony-X peptide synthesizer (Protein Technologies, Inc., Tucson, AZ, USA) using 9-fluorenylmethyloxycarbonyl (Fmoc) chemistry [31,37,38]. The bifunctional NOTA chelator (1,4,7-triazacyclononane-1,4,7-triacetic acid) was conjugated to the N-terminal of the deacetylated R954 sequence, which had been previously extended with a non-natural, non-cleavable β-Ala linker residue to mitigate steric hindrance in B1R ligand binding [38]. The purity and identity of purified peptides were evaluated using ultraperformance liquid chromatography–tandem mass spectrometry (UPLC-UV-MS, Waters AQUITY-H-Class-SQD2; Waters Corporation, Milford, MA, USA) (see Supplementary Figure S3 for HPLC and mass spectrometry chromatograms/spectra of the synthesized peptides R954 and NOTA-βAla-R954). Abbreviations for amino acids follow the recommendations of the IUPAC-IUB Commission on Biochemical Nomenclature. Other abbreviations are described as follows: Orn, l-ornithine; Oic, l-(2S,3aS,7aS)-octahydro-1H-indol-2-carboxylic acid; (αMe)Phe, α-methyl-l-phenylalanine; β-Nal, 3-(2-naphthyl)-l-alanine.

### 2.10. Labeling of NOTA–Peptides with ^64^Copper (^64^Cu) or Natural Cu (^Nat^Cu)

The NOTA–peptide conjugate was next labeled with ^64^Cu under previously optimized conditions [39,40]. Briefly, the NOTA-βAla-R954 (3–5 nmol) was incubated with [^64^Cu]Cu(OAc)_2_ (240–450 MBq) in 0.1 M ammonium acetate buffer pH 5.5 in a total volume of 300–400 μL. The resulting solution was incubated at room temperature for 15–20 min. The radio chemical yield (RCY > 99%) was determined by radio-TLC on C18 plates with 0.1 M sodium citrate buffer (pH 5.5) as the mobile phase; conditions were optimized to achieve quantitative labeling without HPLC or C18 purification. The amount of radioactivity of labeled peptides was measured using a Capintec radioisotope dose calibrator (Capintec, Inc., Florham Park, NJ, USA). The resulting ^64^Cu-peptides were reconstituted in phosphate-buffered saline (PBS, pH 7.4) for in vitro studies or in isotonic saline for in vivo studies.

For labeling with ^Nat^Cu, 2 mg of the NOTA-conjugated peptide was resuspended in 200 µL of a 0.1 M ammonium acetate in water/ACN (1:1). Three equivalents of Cu(OAc)_2_ in water (0.03 M) were added, and the mixture was incubated at room temperature for 30 min. ULPC-MS analysis confirmed the incorporation of copper in the NOTA moiety of the peptide (+61, addition of copper minus two hydrogen). The reaction mixture was then lyophilised and purified using solid-phase extraction (SPE) columns to remove excess copper. The SPE procedure involved conditioning the columns with acetonitrile (ACN) twice, equilibration with water containing 0.1% TFA, applying the peptide dissolved in 0.1% TFA, washing with water containing 0.1% TFA, and eluting with 75% ACN in water containing 0.1% TFA. The collected eluate was lyophilized and stored at −20 °C until further use.

### 2.11. Generation of Stable Human Kinin B1R-HEK293 Cell Line

The B1R minigene (NM_000710) was synthesized (Integrated DNA Technologies, Coralville, IA, USA) and cloned into the lentiviral vector pCDH-CMV-MCS-EF1-puro (System Biosciences, Palo Alto, CA, USA) using BamHI and NotI restriction enzymes (New England Biolab, Ipswich, MA, USA). Lentiviruses were produced by co-transfecting HEK293T cells with the lentiviral vector containing the human B1R gene (hB1R) (pCDH-CMV-B1R-MCS-EF1-puro), the psPAX2 packaging plasmid (a gift from Didier Trono, Addgene plasmid #12260), and the pMD2.G plasmid expressing VSV-G (a gift from Didier Trono, Addgene plasmid #12259) using PEI (Sigma-Aldrich) as the transfection reagent. Lentiviruses were harvested 48 h post-transfection and used to infect QBI HEK293A cells (ATCC). Transduced cells were selected using 1 µg/mL of puromycin (Thermo Fisher Scientific). B1R mRNA expression in the transduced cells was confirmed by radioligand binding studies using [^3^H]-LysdesArg^9^BK, (ART2475; 90–120 Ci/mmol; American Radiolabeled Chemicals Inc., St. Louis, MO, USA), yielding a Kd value of 1.3 nM and a Bmax of 181,000 sites/cell (corresponding to about 1.24 pmol/mg protein). The generation of stably transfected human kinin B2R-HEK293 cell lines used in this study has been described in detail elsewhere [37].

### 2.12. Ligand Competition Binding Assays

Radiolabeled ligand competition binding assays were performed on HEK293 cells stably expressing hB1R or hB2R, as previously described [37,38]. Cells were cultured in 24-well plates and incubated with 4 nM [^3^H]Lys[Leu^8^]desArg^9^BK (for B1R studies) (ART1583; 90–120 Ci/mmol; American Radiolabeled Chemicals) or 1 nM [^3^H]BK (for B2R studies) (NET706; 109 Ci/mmol; Perkin-Elmer, Woodbridge, ON, Canada) in serum-free DMEM for 1–2 h in the absence or presence of increasing concentrations of competitors (10^−11^–10^−5^M). Radioactivity in the samples was measured using a β-scintillation counter (Tri-Carb 2800; Perkin-Elmer). Binding affinities were expressed as IC_50_ values, calculated using GraphPad Prism version 8.2.1 software (GraphPad Software, San Diego, CA, USA).

### 2.13. Generation of NFAT-eGFP Reporter Cells

The pLenti-6V5-NFAT-eGFP vector was generated from the pSIRV-NFAT-eGFP vector (a gift from Peter Steinberger, Addgene plasmid #118031). Briefly, NFAT-eGFP gene was PCR amplified using the following primers: 5′-TTTTATCGATTCCTCTAGACTGCCGGATC-3′ and 5′-AAAACCGGTCGATCGACCACTGTGC-3′). The amplified gene was then ligated into the ClaI and AgeI restriction sites of the pLenti-6V5 vector. Lentiviruses were produced using the pLenti-6V5-NFAT-eGFP vector in HEK293T cells and used to transduce a hB1R-expressing CHO cell line, followed by selection with 10 µg/mL blasticidin. The stable cell population was treated with 1 μM of LysdesArg^9^BK (LDBK; reference agonist of hB1R) [41,42] for 24 h and then sorted for high fluorescence expression using a Sony SH800S cell sorter (Sony Biotechnology, San Jose, CA, USA). Clones were grown in 4000 nanowells/well in 24-well plates and re-plated into 96-well plates after initial expansion via the ALS CellCelector™ (Sartorius Corporation, Bohemia, NY, USA). After clonal expansion, individual cell lines were evaluated based on their ratio of stimulated fluorescence to basal expression to determine maximum sensitivity. The most effective clone was retained as the hB1R reporter cell line.

### 2.14. NFAT-eGFP-Based Reporter Assays

Fluorescence assays performed on CHO-hB1R-NFAT-eGFP cells. Target cells were plated at 10,000 cells/well in a 96-well microplate (opaque black wells with clear flat bottom, Thermo Scientific, Rockford, IL, USA) using RPMI 1640 medium with 10% fetal bovine serum (FBS). After 24 h, the medium was replaced by RPMI without FBS and phenol red. Cells were then stimulated with the B1R agonist LDBK at concentrations ranging from 1 pM to 10 μM at 37 °C/5% CO_2_ for 16 h, in the presence or absence of 500 nM antagonists (preincubated for 10 min prior to agonist addition). Prolonged stimulation times (24 h and 48 h) did not result in increased EGFP signal levels. Fluorescence was measured using a TECAN M1000 (Tecan Group Ltd., Männedorf, Switzerland) multimode microplate reader with excitation at 488 nm and emission at 507 nm. Each data point represents a ratio of two values, divided by the average fluorescence of six control wells. The potencies of the agonists LDBK (EC_50_ value: 2.1 nM) and desArg^9^-BK (DBK: EC_50_ value: 297 nM), consistent with the expected pharmacological profiles on hB1R [42], were calculated by curve-fitting using GraphPad Prism 8.2.1 (GraphPad Software) (Supplementary Table S2). Antagonist affinity (Kb) was estimated from functional inhibition curves using the Gaddum (Schild) equation assuming competitive antagonism at human B1R: Kb = [B]/(dose-ratio–1), where [B] is the antagonist concentration, and the dose ratio is EC_50_ + Compound/EC_50_.

### 2.15. Human Vein Contractility Assays

The potency (apparent affinity) of non-radioactive NOTA-βAla-R954 peptide conjugates at native inducible B1R was assessed using human tissue bioassays with isolated umbilical veins, as previously described [41,43]. Collection of umbilical cords for these bioassays was approved by the CHUS Human Research Ethics Committee (protocol #97-14; 21 March 1997), and maternal consent was obtained in all cases. Antagonist affinity was expressed in terms of IC_50_, which represents the molar concentration of an antagonist that reduces the response to a submaximal concentration of the DBK agonist by 50%.

### 2.16. Ex Vivo/In Vivo Blood Stability Assays

Experiments were performed according to previously published procedures [39,40]. Briefly, aliquots (20 µL) of radiolabeled R954 peptide conjugate (110–185 MBq; 3–5 mCi) were mixed with 250 µL of undiluted plasma collected from healthy Balb/C mice or human male volunteers and incubated in a 37 °C water bath for 24 h. After incubation, the samples were treated with acetonitrile at a 1:1 ratio, followed by centrifugation at 13,000× *g* for 15 min to precipitate and remove plasma proteins. A 100 µL aliquot was taken from the supernatant and analyzed using a Waters^®^ ACQUITY UPLC^®^ H-Class Bio System equipped with an ELSD detector (Waters Corporation) and a BioScan Flow Count radiodetector (BioScan Inc., Washington DC, USA).

In vivo stability assays were carried out following bolus intravenous (IV) injection of radiolabeled R954 peptide conjugate (54 MBq; 1.5 mCi; 200 μL) into the tail vein of air/isoflurane-anesthetized normal Balb/C mice. Terminal blood and urine samples were collected 90 min post-injection from anesthetized animals via carotid puncture (blood) and bladder puncture (urine). Blood samples were centrifuged at 2000× *g* for 15 min at 4 °C, and the resulting plasma supernatants were processed as described above for UPLC analyses. The integrity of the radiolabeled peptide was assessed by comparing the retention times of plasma and urine samples to those of the control, unprocessed radiolabeled peptide.

### 2.17. Cellular Uptake and Nuclear Localization

Cellular uptake profile of ^64^Cu-labeled R954 peptide conjugate was evaluated in PCa cells as described [39,40], with some modifications. Cells were seeded into 6-well plates (2.5 × 10^5^ cells/well) 48 h before the beginning of experiments. Cells were then incubated in fresh media with ^64^Cu-labeled R954 (10 kBq; ~500 nM final) at 37 °C for 15 min, 4 h, or 24 h. For determination of specific internalization, one set of wells was preincubated with unlabeled free R954 (1 µM) at 37 °C for 10 min to block B1R prior to incubation with ^64^Cu-labeled R954. At each time point, radioactive media was collected, and wells were successively washed, once with Hank’s balanced salt solution (HBSS), treated with 50 mM CH_3_COOH/250 mM NaCl (pH 2.5) for 3 min (to remove surface-bound fraction), and twice with PBS, pH 7.4. Thereafter, cells were gently harvested with Gibco’s StemPro Accutase (Life Technologies Corp, Grand Island, NY, USA). In some experiments, PCa-derived nuclei were also isolated after the indicated time point using the procedures described in the following section. ^64^Cu activity in the media, intact cells, and isolated nuclei was then measured with a gamma counter (Cobra II auto-gamma counter, Packard, Minneapolis, MN, USA). The percentage of radioactivity in the nucleus was determined as the ratio of gamma counts in the pure nuclei over gamma counts associated with whole cells. Precise cell/nucleus counts (with Trypan blue staining) were determined on mirror plates following cell detachment or nuclei isolation procedure.

### 2.18. Cell Fractionation and Nuclei Isolation

PCa cells were grown on 100 mm dishes at confluency. Following treatment with ^64^Cu-labeled R954, adherent cells were washed once in Hank’s balanced salt solution (HBSS) then subjected to an acid wash (50 mM acetic acid/250mM NaCl at room temperature) for 3 min to remove any residual membrane-bound radiolabeled peptides. Cells were next washed twice with PBS, detached using StemPro Accutase^®^ solution (Life Technologies), washed once more with PBS, and then counted. Cell nuclei were isolated according to Wang et al. [44] with some modifications. Briefly, cell pellets were resuspended in hypotonic lysis buffer solution (10 mM PIPES pH 6.8, containing 100 mM NaCl, 2 mM MgCl_2_, 300 mM sucrose, and 0.5% Triton X-100) and allowed to rest on ice for 3 min. Samples were then diluted ten times with hypotonic buffer without Triton X-100, centrifuged at 700× *g* for 5 min. The resulting supernatant (cytoplasmic fraction) was then collected while the pellet (nuclear fraction) was resuspended in detergent-free hypotonic buffer. Total proteins in cytoplasmic and nuclear extracts were measured using BCA™ (bicinchoninic acid) protein assay kit (Thermo Scientific). Purity of nuclear fractions were assessed by light microscopy after trypan blue staining and by Western blotting with a set of antibodies against specific organelle markers: lamin A/C and nuclear pore complex (nucleus), clathrin and Na^+^/K^+^ ATPase (cell membrane), calnexin (endoplasmic reticulum), LAMP-2 (lysosome), cytochrome C (mitochondria), and tubulin (cytosol) (see text below).

### 2.19. Western Blotting

Western blotting was performed on 25–50 µg of heat-denatured protein extracts, prepared by homogenization in conventional RIPA buffer containing a protease inhibitor cocktail (Sigma-Aldrich), migrated through a 9–12% acrylamide gradient SDS-PAGE Tris-Glycine gel (Invitrogen), and electroblotted onto PVDF membranes. Blots were blocked using 5% non-fat dry milk and probed using appropriate primary antibodies in blocking buffer overnight at 4 °C. Proteins were visualized using a goat anti-rabbit (1:10,000) or anti-mouse (1:20,000) secondary antibody conjugated to enzyme horseradish peroxidase (HRP) and a western lightning chemiluminescence detection kit, as per manufacturer’s instructions (PerkinElmer), and revealed by use of X-Omat film (Eastman Kodak Company, Rochester, NY, USA).

### 2.20. Intracellular Copper Measurement by Inductively Coupled Plasma Mass Spectrometry (ICP-MS)

PCa cells were seeded in 10 cm plates and after reaching ~90% of confluency they were exposed or not to non-radioactive ^Nat^Cu/NOTA-βAla-R954 for 24 h at 37 °C in RPMI 1640 media with 1% FBS. Cells were washed once in HBSS then subjected to an acid wash as above mentioned. Thereafter, cells were washed twice with PBS, harvested using StemPro Accutase^®^ solution (Life Technologies), centrifuged twice in PBS, and then counted. Cell nuclei were isolated as described above (Section 2.18). Cell pellets and nuclei were resuspended and digested overnight at 37 °C in a 50:50 mixture of concentrated HNO_3_/30% H_2_O_2_. Dissolved Cu was measured with inductively coupled plasma mass spectrometry (ICP-MS; Perkin-Elmer Elan DRC II). Samples were diluted with nanopure H_2_O to a final concentration of 2% nitric acid/0.4% H_2_O_2_ solution (*v*/*v*) before Cu measurements. Cu calibration standards (from 0.1 to 50 ppb) were prepared in the same 2% nitric acid/0.4% H_2_O_2_ solution.

### 2.21. Clonogenic Assays

PCa cells were seeded in 6-well plates (at ~400 cells/well) in RPMI medium supplemented with 10% FBS and treated 24 h later with varying doses of either the ^64^Cu-labeled R954 conjugate or its ^Nat^Cu counterpart. The plates were incubated at 37 °C for approximately 10 to 14 days, before staining and counting [45]. To prevent possible bystander irradiating effects from occurring, cells were seeded in one well on two opposite sides of 6-well dishes.

### 2.22. Subcutaneous Xenograft Models of Human PCa

Male Nu/Nu nude mice (Crl:NU-Foxn1nu: 4–6 weeks old, Charles River Laboratories, Inc., Wilmington, MA, USA) were subcutaneously (s.c.) inoculated into one or both lower flanks with PCa cells. LNCap cells (10 x 10^6^) and PC3 cells (2.5 × 10^6^) were suspended in 0.2 mL of a 1:1 (*v*/*v*) mixture of antibiotic-free DMEM medium and Matrigel (#356234, Sigma-Aldrich) for injection. Within 10–21 days post-inoculation, solid palpable prostate tumors had developed, and the animals were used for biodistribution, PET imaging, and radiotherapy studies. All animal experimentation was approved by the Animal Care Committee (CPA) of the Faculty of Medicine and Health Sciences (FMSS), Université de Sherbrooke (protocols #392-15BR, 14 July 2015; #2019-2371, 5 March 2020) and conducted in accordance with the guidelines of Canadian Council on Animal Care.

### 2.23. Biodistribution Studies and Micro-PET Imaging

To perform ex vivo biodistribution studies, radiolabeled peptides (5–10 MBq; 100 μL) were administered to isoflurane-anaesthetized normal male Balb/c mice through caudal vein injection. Organs were collected 1 h and 4 h post-injections from CO_2_ inhalation-euthanized animals and were washed, weighted, and radioactivity measured in a gamma counter (Cobra II auto-gamma counter, Packard). The percentage of injected dose per gram of tissue (%ID/g) was then calculated, accounting for decay correction, using external ^64^Cu standards. Biodistribution of the radiolabeled peptides was also performed in PCa tumor-bearing nude mice using micro-PET imaging performed on a 7.5 cm axial and 10 cm transaxial field of view LabPET8 small-animal scanner (Gamma Medica-IDEAS Inc., Northridge, CA, USA) in combination with X-ray computer tomography (CT) (MILabs U-CT system, Utrecht, The Netherlands) [46]. For this, radiolabeled peptides (6–9 MBq in 200 μL) were delivered by tail-vein injection to isoflurane-anaesthetized mice. PET scans composed of 30 min static acquisition were acquired at 1 h and 20 h after injection. Using the PMOD software (version 3.8, PMOD Technologies Ltd., Zurich, Switzerland), the PET image of each animal was co-registered with the corresponding CT image by a rigid matching transformation. PET images were converted to a percentage of injected dose per gram (%ID/g) using a calibration phantom loaded with known activity concentration [47]. To quantify the tumor uptake of the radiotracer, a volume of interest (VOI) was drawn around the center of the tumor. The uptake was determined as the means of the VOI voxels having intensity in the upper quartile.

### 2.24. Peptide Receptor Radionuclide Therapy (PRRT)

Therapeutic efficacies of agents were investigated in PC3 xenograft-bearing mice. Once tumors reached a size of 50–200 mm^3^, mice were randomly assigned to three groups and treated via tail-vein injection with either a single equivalent dose of ^64^Cu/NOTA-βAla-R954 (50 µg/kg; 65 MBq or 1.75 mCi), its non-radioactive counterpart ^Nat^Cu/NOTA-βAla-R954 (50 µg/kg), or an equal volume of control vehicle (100 µL isotonic saline), under mild isoflurane anesthesia. Although no dosimetric analysis was performed, the selected radiation dose was guided by previously published studies and was within the established range commonly used for ^64^Cu-based radiotherapy applications [48,49,50,51]. Tumor growth was monitored with a digital vernier caliper using the following formula: length × width^2^. The weight of the mice was monitored throughout the study. The first day of drug administration was designated as day 0. Tumor growth was normalized by calculating the ratio of the tumor volume at each time point to the initial tumor volume measured prior to treatment. The weight of the mice was monitored throughout the study. At the end of experiments, all animals were euthanized by CO_2_ asphyxiation and tumors were then totally excised, photographed, and FFPE blocks of tumor tissues prepared for IHC biomarker analyses.

### 2.25. IHC Staining of Resected PCa Xenografts

Retrieved subcutaneous tumors (5 μm thick sections) were evaluated by H&E and semiquantitative IHC staining of B1R, Ki67 (proliferation), cleaved caspase 3 (apoptosis), CD34 (angiogenesis), and LYVE-1 (lymphangiogenesis). An isotype-matched rabbit-/mouse-IgG (Dako, Hamburg, Germany), instead of the primary antibody, served as negative controls (see Supplementary Figure S2). Multiple fields of view were imaged for experimental groups to ensure adequate representation of the whole tumors. Whole slides were digitized using Hamamatsu NanoZoomer scanning system (Hamamatsu Corporation) and quantitative image analysis carried out using Image-Pro Plus (version 5.1; Media Cybernetics, Rockville, MD, USA) or Nanozoomer Digital Pathology (NDP) view 2 imaging software (version 2.6.13; Hamamatsu Corporation). For the analysis of each IHC-stained tissue section, an average of 3–5 fields per tumor (3–4 tumors per group), excluding necrotic zones, were randomly sampled (under 10 to 40× magnification). For Ki67, only nuclear immunoreactivity was considered specific, as recommended in the literature [52]. Positive Ki67 staining was determined by the nucleus count (Ki-67+ cells/total cells) in sampled fields. CD34 or LYVE-1-positive microvessel number were counted and reported over the total area (in mm^2^) used for counting (calculated using Nanozoomer Digital Pathology software version 2.6.13). Quantification of active caspase-3 and B1R-positive immunostaining was achieved by dividing the number of positive pixels by the total pixel count in each selected field. For B1R, calculated values from treated specimens were normalized to those of untreated control tumors. Selection and discrimination between positive and negative pixels were performed using the color segmentation tool of Image-Pro plus software (version 5.1; Media Cybernetics).

### 2.26. Statistical Analysis

Data are expressed as the mean ± sem. Where appropriate, the data were analyzed by either one-way ANOVA followed by Dunnett’s test, Tukey’s multiple comparison test, or unpaired Student’s *t*-test (GraphPad Prism v10.2). *p* values < 0.05 were considered statistically significant.

## 3. Results

### 3.1. Expression of B1R in Human PCa Cell Lines and Tumors

**Figure 1:**
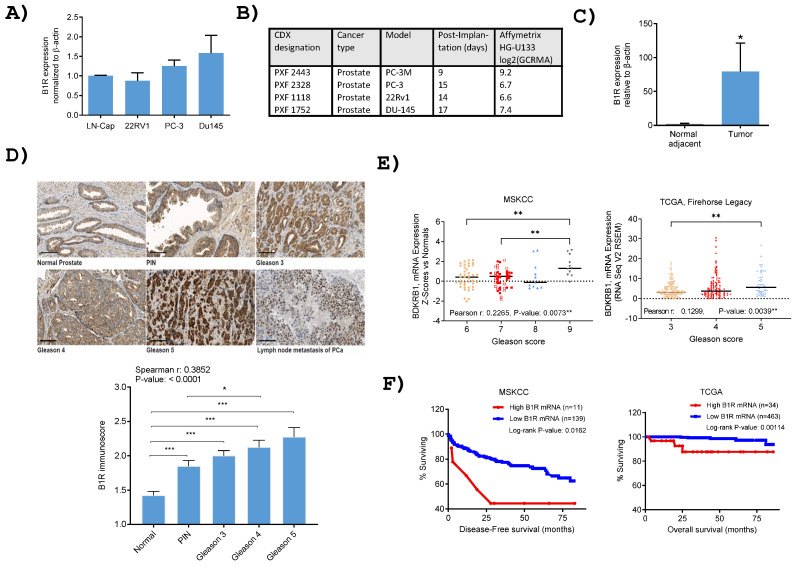
(**A**) Expression of B1R in human PCa cell lines assessed by qPCR (*n* = 2–3). (**B**) B1R gene expression analysis of the human PCa cell line-derived xenografts (CDX) in Nu/Nu mice (four CDX models from Charles River). Affymetrix data are presented as log2 (GCRMA), with higher values indicating increased B1R expression. (**C**) B1R expression in clinical human PCa tissue samples (tumor and matched normal-adjacent; *n* = 14) analyzed by qPCR. * Indicates *p*-value < 0.05 according to paired Student *t*-test. (**D**) Representative pictures taken from cores of normal, PIN, and Gleason 3–4–5 foci, and of metastatic PCa in peripancreatic ganglia. Magnification 20×, scale bar represents 100 µm. Semi-quantitative immunoscores (as per scoring scale shown in Supplementary Figure S2) of normal (*n* = 95), PIN (*n* = 71), Gleason 3 (*n* = 126), 4 (*n* = 64), 5 (*n* = 18), * indicates *p*-value < 0.05 and *** <0.001 according to a one-way ANOVA with Tukey’s multiple comparison tests. (**E**) Histogram views and Pearson r correlation coefficients of hB1R gene expression according to tumor Gleason score from The Cancer Genome Atlas (TCGA) firehose legacy and the Memorial Sloan Kettering Cancer Center (MSKCC) 2010 datasets, both retrieved using cBioportal tool. ** Indicates *p*-value < 0.01 according to Tukey’s multiple comparison tests. (**F**) Kaplan–Meier survival curves according to prostate cancer B1R (BDKRB1) mRNA expression levels for MSKCC Cancer Cell 2010 dataset (high and low, respectively > or <z-score 2.25 compared to normal tissues) and TCGA Firehose Legacy (high and low, respectively > or <z-score 1.75 vs. diploids) accessed by cBioportal. Log-rank test *p*-values are indicated within each graph.

RT-qPCR analysis demonstrated comparable levels of B1R expression across all tested human PCa cell lines (LNCaP, PC3, 22RV1, DU145; Figure 1A). Among them, LNCaP and PC3 exhibited cell surface B1R expression, as evidenced by FACS analysis (Supplementary Figure S4). Additionally, B1R gene expression analysis in four human PCa cell line-derived xenografts (CDXs) implanted in Nu/Nu mice revealed moderate variability, with log2 values ranging from 6.6 to 9.2 across the xenografts (Figure 1B). In a series of PCa tumors and paired adjacent non-cancerous tissues (*n* = 14), B1R mRNA expression levels were significantly overexpressed, with increases of up to 40-fold (*p*-value: < 0.05, Figure 1C). Semi-quantitative assessment of B1R protein levels using PCa tissues microarrays (Figure 1D) showed significantly higher expression in tumors compared to normal prostate glands, with protein levels positively correlating with Gleason scores (Spearman r: 0.385, *p*-value: < 0.0001). Notably, B1R positivity remained strong in one metastatic PCa case (ganglia) examined. These findings were further supported by analyses of two independent datasets retrieved from cBioPortal (MSKCC and TCGA Firehose Legacy), which also revealed positive correlations between B1R mRNA expression and tumor Gleason scores (Figure 1E). Furthermore, Kaplan–Meier survival analyses using the MSKCC and TCGA databases indicate that elevated B1R expression is associated with poor survival outcomes in PCa patients (Figure 1F). Altogether, these findings indicate that B1R overexpression contributes to PCa progression, underscoring its potential as both a biomarker and a therapeutic target, positioning it as a promising candidate for theranostics.

### 3.2. Synthesis, ^64^Cu Radiolabeling, and Stability of a New NOTA-Conjugated Analog of R954 for Theranostic Applications

**Figure 2:**
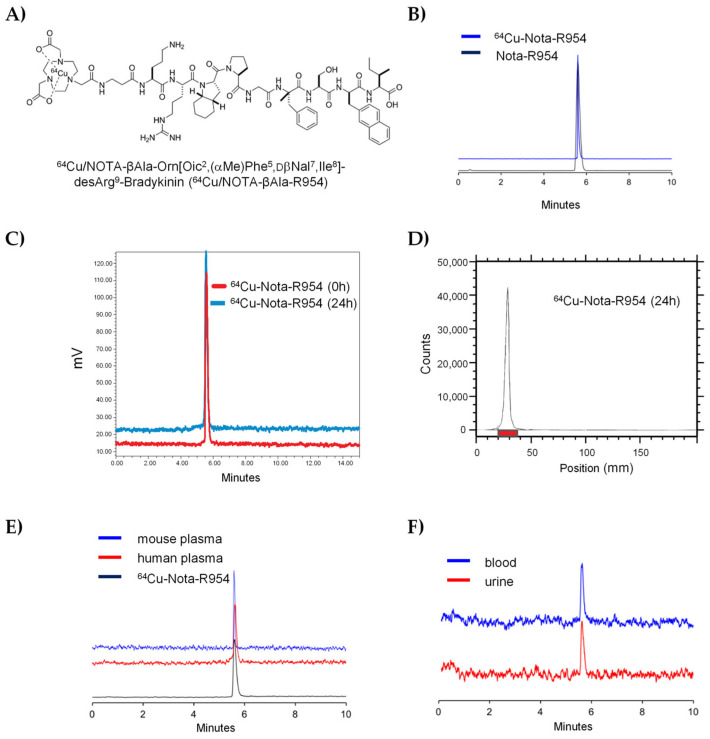
Chemical structure and metabolic stability of the novel B1R-targeting theranostic antagonist, ^64^Cu/NOTA-βAla-R954. (**A**) Theranostic peptide sequence. (**B**) Radiolabeling of NOTA-βAla-R954 to ^64^Cu. Representative radio-UPLC chromatograms are shown. (**C**,**D**) Radiolysis evaluation of ^64^Cu/NOTA-βAla-R954 at time point 0 and 24 h assessed by radio-UPLC and radio-TLC, respectively. Representative experiments are shown. (**E**) Ex vivo metabolic stability of ^64^Cu/NOTA-βAla-R954 following a 24 h incubation at 37 °C in undiluted mouse and human plasma. Representative radio-UPLC chromatograms are shown (*n* = 2). (**F**) UPLC radiometric chromatogram illustrating the complete in vivo stability of the ^64^Cu/NOTA-βAla-R954 (appearing as an intact form—single peak—in blood and urine extracts) following I.V. injection in normal mice. Blood and urine were collected 90 min after bolus I.V. administration (*n* = 2).

The theranostic peptide sequence based on the B1R antagonist R954, and previously reported in our earlier preliminary study [53], is depicted in Figure 2A. The synthesis of NOTA-βAla-R954 and its parent peptide R954 was straightforward, achieving purities exceeding 98%, as confirmed by HPLC and mass spectrometry (NOTA-βAla-R954: mw = 1508.8; R954: mw = 1194.5). Radiolabeling of NOTA-βAla-R954 with ^64^Cu produced a peptide with an apparent molar activity of 104.7 ± 7.6 MBq/nmol (equivalent to 69.4 MBq/µg peptide) and a radiochemical purity greater than 98% (Figure 2B). Stability studies show no evidence of peptide radiolysis observed over a 24 h period, as confirmed by radio-UPLC and radio-TLC analyses (Figure 2C and 2D, respectively). Ex vivo blood stability assessments reveal that the ^64^Cu/NOTA-βAla-R954 exhibited exceptional stability, with no signs of degradation or transmetallation in undiluted mouse and human plasma after a 20 h incubation period (Figure 2E). Furthermore, in vivo studies confirmed the presence of predominantly intact radiolabeled peptides in mouse blood circulation and urine 90 min after intravenous injection (Figure 2F).

### 3.3. Competitive Binding Affinity, Selectivity, and Potency Assays at Recombinant and Native hB1R and hB2R

The results of the pharmacological evaluation of ^Nat^Cu/NOTA-βAla-R954, conducted through multiple in vitro assays, are summarized in Table 1. Binding experiments conducted with HEK293 cells stably expressing hB1R or hB2R demonstrate that the peptide conjugate, whether labeled with Cu or not, retained strong nanomolar affinity for hB1R, albeit slightly lower than its parent peptide R954 (see IC_50_ values). As expected from the properties of the parent molecule, no detectable binding interaction was observed with hB2R (IC_50_ values > 10 µM), confirming its high selectivity. At the cellular level, the peptide conjugates effectively antagonized NFAT luciferase-eGFP reporter activity stimulated by the B1R agonist LDBK, exhibiting low Kb values ranging from 0.4 to 2.2 nM, comparable to that of the unconjugated R954. At the tissue level, ex vivo functional studies using the contraction of isolated human vein vessels show that NOTA covalent conjugation on R954 did not compromise its antagonist potency against native hB1R, while consistently remaining inactive on hB2R.

### 3.4. In Vitro Cellular Uptake, Nuclear Distribution, and Anticancer Activity of ^64^Cu/NOTA-βAla-R954 in PCa Cells

**Figure 3:**
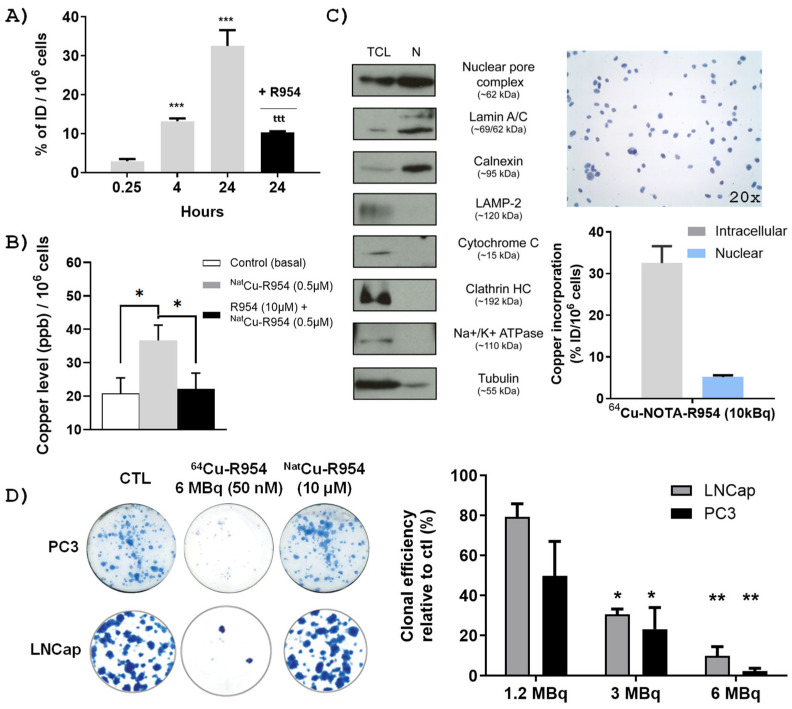
Cellular uptake, nuclear distribution, and anticancer activity of ^64^Cu/NOTA-βAla-R954 in PCa cells. (**A**) Time-course analysis of ^64^Cu-NOTA-βAla-R954 (10 KBq; 0.5 µM) incorporation by PCa PC3 cells. *** *p*-value <0.001 vs. 0.25 h timepoint, according to multiple *t*-tests, *n* = 4; ^ttt^ *p*-value < 0.001 vs. 24 h time-point; unpaired Student’s *t*-tests. (**B**) Cell uptake of ^Nat^Cu-NOTA-βAla-R954 (0.5 µM) in PC3 cells at 24 h post-treatment, as determined by ICP-MS. * *p* < 0.05 according to multiple *t*-tests (*n* = 7–8 assays). (**C**) Intracellular/nuclear distribution of ^64^Cu-NOTA-βAla-R954 (10 KBq; 0.5 µM) in PC3 cells at 24 h post-treatment (*n* = 4 experiments). Validation of the purity of nuclear fractions extracted from PC3 cells by immunoblotting using organelle-specific marker proteins (**left**) and by optical microscopy after trypan blue staining (**upper right**). (**D**) Dose-dependent antiproliferative effects of ^64^Cu/NOTA-βAla-R954 on PCa LNCaP and PC3 cells using clonogenic assays. * *p* < 0.05, ** *p* < 0.01 vs. control, according to one-way ANOVA followed by Dunnett’s multiple comparison, *n* = 4 experiments. No inhibitory effects of the non-radioactive ^Nat^Cu/NOTA-βAla-R954 (10 µM) on PC3 and LNCap cell growth and clonogenic potential.

Cellular uptake is a prerequisite for the effective use of ^64^Cu-labeled agents in biomedical fields, such as PET imaging and PRRT. The data presented in Figure 3 provide insights into the cellular uptake dynamics, nuclear localization, and anticancer activity of ^64^Cu/NOTA-βAla-R954 in PCa cells. Radiometric analysis demonstrate a time-dependent uptake of ^64^Cu/NOTA-βAla-R954, reaching 33% of the injected dose (ID) at 24 h in PC3 cells. Pre-treatment with an excess of B1R antagonist R954 significantly reduced this uptake, confirming the receptor specificity of the process (Figure 3A). These findings were further corroborated through inductively coupled plasma mass spectrometry (ICP-MS) analysis, utilizing the ^Nat^Cu-labeled form of the peptide (Figure 3B).

The delivery of ^64^Cu-labeled peptides to the cell nuclei is known to boost their therapeutic efficacy by emitting Auger electrons, which cause localized DNA damage [30]. To investigate the nuclear distribution of the ^64^Cu/NOTA-βAla-R954, PC3 cells were incubated with the compound for 24 h, followed by subcellular fractionation. The high purity of the nuclear fractions was validated through optical microscopy, which confirmed the absence of intact cells, and by Western blot analysis (Figure 3C). The Western blot results confirm the presence of established nuclear protein markers lamin A/C and nucleoporin, while markers of other subcellular compartments, including LAMP-2 (lysosomes), cytochrome C (mitochondria), clathrin (endosomes), Na^+^/K^+^ATPase (plasma membrane), and tubulin (cytoskeleton), were absent. The detection of calnexin was consistent with its expected localization, as the rough endoplasmic reticulum is contiguous with the nuclear envelope. Quantitative analysis reveals that 5% of the ID of ^64^Cu-labeled peptide—equating to approximately 16% of total intracellular uptake—was localized within or had reached the nuclear compartment 24 h post-incubation (Figure 3C).

The dose-dependent cytotoxic activity of the ^64^Cu-labeled B1R peptide, compared to the control non-radioactive B1R peptide similarly labeled with ^Nat^Cu, was evaluated using clonogenic assays (Figure 3D). Results show that ^64^Cu/NOTA-βAla-R954 effectively suppressed clonogenic growth in both LNCaP and PC3 cell lines in a dose-dependent manner, whereas the nonradioactive counterpart, ^Nat^Cu/NOTA-βAla-R954, even at a much higher concentration (10 µM), had no effect on their clonogenic potential.

### 3.5. In Vivo Biodistribution and Micro-PET Imaging of ^64^Cu/NOTA-βAla-R954

**Figure 4:**
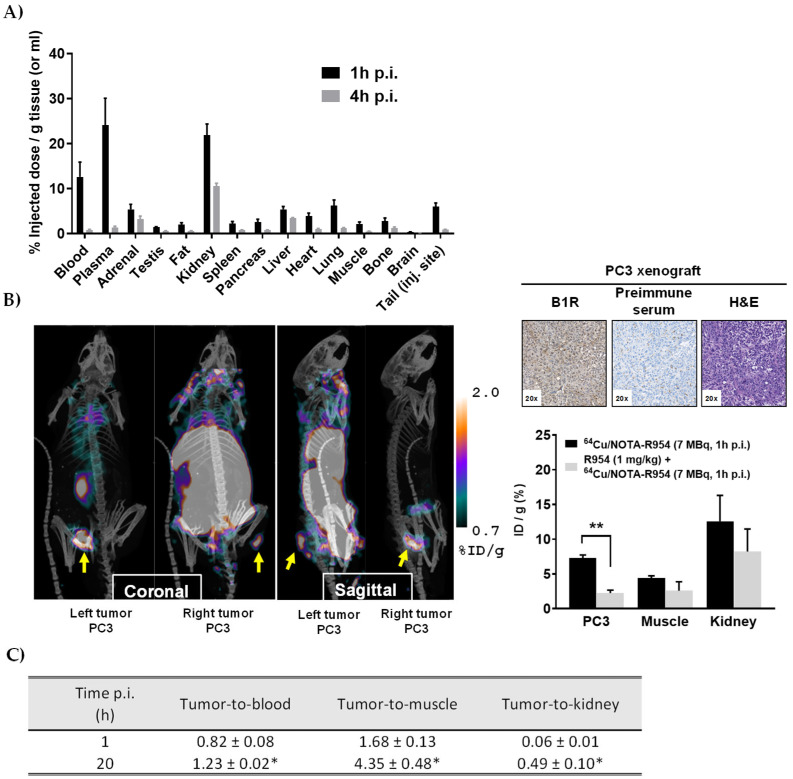
Efficacy of ^64^Cu/NOTA-βAla-R954 for detecting PCa by PET imaging. (**A**) Biodistribution of intravenous ^64^Cu/NOTA-βAla-R954 (5–10 MBq) in normal male BALB/c mice at 1 and 4 h post-injection (p.i.) (*n* = 4 mice per group). (**B**) Representative micro-PET images of PC3 tumor-bearing nude mouse acquired 1 h post-injection of ^64^Cu/NOTA-βAla-R954 (7 MBq) using a LabPET8 scanner (**left**). Tumors are indicated by yellow arrows. Histographic representation of calculated ID/g (%) values of intravenous ^64^Cu/NOTA-βAla-R954 (6–7 MBq) at 1 h post-injection in PC3 tumor-bearing nude mice pretreated (10 min before) or not with R954 (0.5–1 mg/kg) (**right**). Mean ± SEM are shown; *n* = 4 tumors from 2 to 3 mice, ** *p* < 0.01 vs. corresponding blockade group, using unpaired two-tailed Student’s *t*-test. IHC confirmed high expression of B1R in harvested tumoral tissues of PC3-bearing mice (**upper-right panel**). IHC staining of B1R was performed on 5 µm thick sections of formalin fixed/paraffin-embedded tissues from subcutaneous PC3 human xenograft tumors in athymic nude mice. IHC analysis was performed using the anti-hB1R antiserum AS434 (1:500 dilution). Negative control with rabbit pre-immune serum (1:500) showed no staining. Nuclei were counterstained with hematoxylin. Optical magnification is indicated in the bottom-left corner of each image. (**C**) PC3 tumor-to-normal-organ ratios for ^64^Cu/NOTA-βAla-R954 (~6–7 MBq) at 1 h and 20 h post-injections. Mean ± SEM are shown; *n* = 4 tumors from 2 to 3 mice. * *p* < 0.05 vs. corresponding ratio at 1 h, using Mann–Whitney U test.

The results of the in vivo tissue biodistribution of IV-administered ^64^Cu/NOTA-βAla-R954 in normal male BALB/c mice, along with the PET imaging findings in male nude mice bearing subcutaneous PCa tumors, are summarized in Figure 4. The results demonstrate rapid clearance of the radioactive peptide from the bloodstream, as observed at 1 h and 4 h post-injection. Significantly higher levels of radioactivity were detected in the kidneys compared to other organs, gradually decreasing over time from 24% to 10% of the injected dose per gram of tissue (ID/g) (Figure 4A). These findings highlight the peptide’s preferential accumulation and excretion through renal pathways, consistent with previously reported observations for the parental peptide R954 [23]. Moreover, PET images acquired 1 h after injection of ^64^Cu/NOTA-βAla-R954 clearly visualized the PC3 tumors located in the flanks (Figure 4B, left panel). The accumulation of the radioconjugate (7 MBq; ~4 µg/kg) in PC3 tumors, as shown by PET imaging, but not in the muscles or kidneys, could be blocked by an excess of unlabeled R954 (1 mg/kg). This demonstrates the peptide’s specificity for B1R-expressing tumors, as confirmed by positive IHC staining of resected tumors at the specified time post-inoculation (Figure 4B, right panel). At 1 and 20 h post-injection, the tumor-to-blood, tumor-to-muscle, and tumor-to-kidney ratios for ^64^Cu/NOTA-βAla-R954 significantly increased, suggesting a preferential retention of the radiotracer in tumor tissues over time compared to other compartments (Figure 4C).

### 3.6. Effects of PRRT with ^64^Cu/NOTA-βAla-R954 on PCa Growth

**Figure 5:**
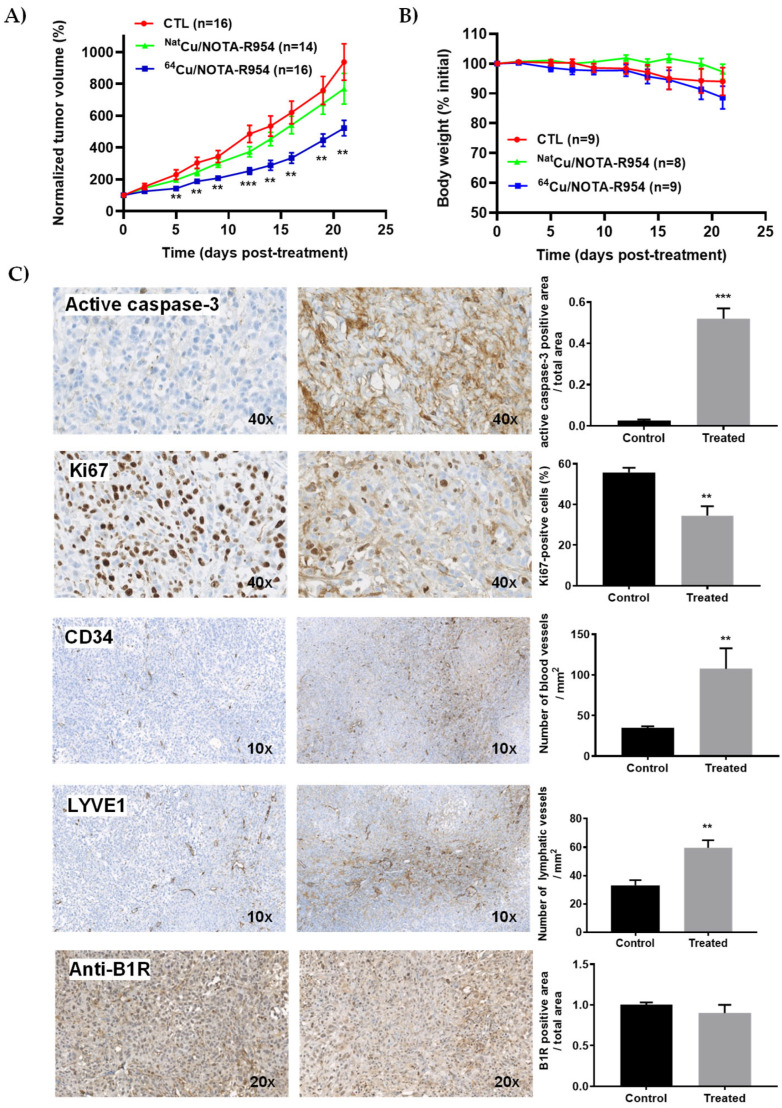
Comparison of the anticancer efficacy of a single-dose IV ^64^Cu/NOTA-βAla-R954 (50 µg/kg; 65 MBq) and ^Nat^Cu/NOTA-βAla-R954 (50 µg/kg) in nude mice bearing s.c. PC3 xenografts. (**A**) Normalized tumor growth following treatment. Data represent the mean ± SEM from 14 to 16 tumors per group (*n* = 8–9 mice/group); ** *p* < 0.01, *** *p* < 0.001 vs. CTL, using unpaired Student’s *t*-test with Welch correction. (**B**) Changes in body weight of animals after treatments. (**C**) Expression levels of active caspase-3 (apoptotic), Ki67 (proliferative), CD34 (angiogenic), and LYVE-1 (lymphatic) biomarkers in PC3 tumors. Representative micrographs of tumor sections are shown for control (saline) and PRRT-treated groups (**left** and **right panels**, respectively). Histograms depict quantitative analysis of marker expression, presented as mean ± SEM from 6 to 8 tumors per group (*n* = 3 mice). ** *p* < 0.01, *** *p* < 0.001 vs. CTL, using unpaired two-tailed Student’s *t*-tests.

The comparison of the anticancer efficacy of an equivalent single-dose IV administration of ^64^Cu/NOTA-βAla-R954 (50 µg/kg; 65 MBq) and ^Nat^Cu/NOTA-βAla-R954 (50 µg/kg) in nude mice bearing s.c. PC3 xenografts is presented in Figure 5. In contrast to the non-radioactive ^Nat^Cu-labeled peptide, the ^64^Cu/NOTA-βAla-R954 conjugate significantly reduced tumor growth (Figure 5A). Notably, the PRRT treatment did not induce any observable toxicity, as indicated by the absence of changes in body weight throughout the study (Figure 5B). This anti-tumor activity was attributed to mechanisms involving cancer cell apoptosis, as indicated by increased expression of active caspase-3, and reduced proliferation, evidenced by the downregulation of Ki67 biomarkers (Figure 5C). Angiogenesis and lymphangiogenesis are key features of PCa progression and metastasis [54]. Therefore, the expression of CD34 and LYVE-1, markers for vascular and lymphatic vessels, respectively, was also assessed in resected prostate tumors from mice treated with or without ^64^Cu/NOTA-βAla-R954 using IHC (Figure 5C). Surprisingly, the numbers of both lymphatic (LYVE-1) and vascular (CD34) vessel counts significantly increased after PRRT treatment, suggesting the activation of a compensatory mechanism that may support tumor survival. Interestingly, PCa tumors remained positive for the hB1R target in the ^64^Cu/NOTA-βAla-R954-treated group (Figure 5C), highlighting the potential to extend the number of PRRT treatment cycles to further improve therapeutic outcomes and possibly achieve complete cancer eradication.

## 4. Discussion

In this study, we demonstrated consistent expression of B1R transcripts across various cultured human PCa cell lines, CDX-derived xenografts implanted in nude mice, and resected human PCa tumor samples. Furthermore, B1R proteins were found to be overexpressed in primary PCa (and potentially in lymphatic metastases), with their expression significantly correlating with higher overall tumor grades. This observation was further validated through analyses of data from the TCGA and MSKCC databases, which also reveal that elevated B1R mRNA levels were associated with poor prognosis and reduced survival rates in PCa patients. Similarly, data from the Human Protein Atlas (HPA) show that high B1R mRNA levels were also linked to decreased survival in lung and kidney cancer patients (see Supplementary Figure S5) [55], suggesting that B1R expression may serve as an unfavorable prognostic marker in various malignancies. Collectively, the findings highlight the potential of B1R as a valuable biomarker for both the detection and therapeutic targeting of PCa. Building on this insight, we set out to develop the first ^64^Cu-based radiotheranostic agent specifically designed to target B1R in PCa, leveraging the peptide antagonist R954. Our B1R antagonist has successfully undergone a rigorous preclinical development program, including a comprehensive evaluation of safety, efficacy (as a potential novel analgesic and anti-inflammatory agent), and ADME profiling, thereby confirming its suitability for clinical translation [23]. Aligned with this objective, we provide supportive evidence that the radiolabeled ^64^Cu/NOTA-βAla-R954 is a promising dual-purpose agent for both imaging and therapy in subcutaneous PCa mouse model with endogenous B1R expression, eliminating the need for radionuclide substitution. This is particularly important because previous studies have shown that switching the coordinating metal from an imaging radionuclide (e.g., ^68^Ga) to a therapeutic one (e.g., ^111^In or ^177^Lu) can drastically alter the pharmacodynamic and pharmacokinetic properties of metalated compounds [56].

Recent advancements have been made in the development of B1R-targeted radiolabeled peptides for PET imaging of cancers (see an excellent review [57]). For instance, Lin’s group reported a series of peptidic B1R radiolabeled agonists and antagonists as novel PET radiotracers, using mice xenografted with HEK293T kidney cells highly overexpressing recombinant hB1R as tumor surrogates (see review [57]). However, many of these B1R radiolabeled peptides have exhibited suboptimal in vitro and in vivo stability, primarily due to degradation by off-target peptidases in blood and tissues, which could contribute to false-positive results. Additionally, the potential formation of toxic radiotracer metabolites could pose a significant concern in theranostic applications, particularly in the context of high-dose radiation therapy with appropriate radionuclides. The same group also utilized the antagonist R954 to develop ^68^Ga and ^18^F-labeled PET agents for non-invasive B1R assessment (^68^Ga-DOTA-dPEG2-R954 and ^18^F-AmBF_3_-Mta-dPEG_2_-R954) in their experimental animal model (see review [57]). Similarly, Shukuri et al. recently evaluated the ^111^In-DOTA-Ahx-R954 in a subcutaneous, natively B1R-expressing U87 brain cancer model in mice as potential PET imaging agents [58]. Both studies yielded consistent positive results in terms of B1R detection [57,58].

In our study, we focused on a B1R-targeting theranostics based on the R954, which displays structural differences from the previously reported compounds, more specifically in the linker, chelator, and radionuclide. As observed in numerous studies on molecularly targeted radiotherapeutics [40,56,59], these structural components can significantly influence the binding affinity, pharmacokinetic profile, and ability to target receptor-expressing tumors in vivo. To assess the impact of these modifications, we first radiolabeled NOTA-βAla-R954 with ^64^Cu, achieving high yield and excellent apparent molar activity. This efficient radiolabeling process ensures the production of a well-characterized tracer for subsequent pharmacological assessments. In vitro, this peptide conjugate retained strong B1R binding affinity and antagonistic potency (in the low nanomolar range), along with robust plasma stability, and significant uptake in PCa cells naturally expressing B1R. A possible mechanistic explanation for this uptake is that B1R antagonists may be passively internalized via the receptor’s constitutive, agonist-independent trafficking [60,61]. Additionally, the B1R radioantagonist ^64^Cu/NOTA-βAla-R954 showed dose-dependent anti-clonal growth effects, irrespective of the androgenic status of PCa cells. In vivo, ^64^Cu/NOTA-βAla-R954 demonstrated exceptional stability in the systemic circulation (up to 90 min), with no detectable free ^64^Cu or metabolites. It also exhibited efficient renal clearance, as evidenced by biodistribution analysis showing no significant accumulation in most organs over time. This observation is in line with the limited B1R expression and minimal functional activity in major organs. Like most radiolabeled peptides cleared via the kidneys, this may pose a risk of nephrotoxicity. However, renoprotective strategies, such as co-administering basic amino acids to reduce kidney retention, have proven effective in clinical PRRT [62,63,64] and could be employed if necessary. Moreover, ^64^Cu/NOTA-βAla-R954 (7.5 MBq IV) effectively detected PCa xenografts in mice, as confirmed by PET/CT imaging. Importantly, at a higher dose (65 MBq or 50 µg/kg IV), it significantly inhibited tumor growth without observable adverse effects, such as weight loss, in PC3 tumor-bearing athymic nude male mice. This anticancer effect was associated with increased apoptosis (evidenced by active caspase-3) and decreased tumor cell proliferation (Ki67), along with potential compensatory angiogenic responses. In contrast, the nonradioactive ^Nat^Cu/NOTA-βAla-R954 showed no effect at the same dose. B1R antagonists have been shown to delay tumor growth in preclinical cancer rodent models including PCa, through direct effects on cancer cells and indirect bystander effects on host stromal cells, leading to increased survival [18,19,20,65]. However, achieving these therapeutic benefits typically requires repeated administration of high doses of these antagonists. Thus, our findings underscore significant differences and distinct advantages in the therapeutic efficacy of specific B1R antagonists when combined with radionuclide therapy.

As part of our B1R-targeted theranostic plan strategy, and for comparative analysis, additional ^64^Cu-labeled NOTA-R954 conjugates were designed, synthesized, and characterized in vitro. Their binding affinity (using B1R-HEK293 cells) and potency (using B1R-CHO-NFAT cells) were assessed under identical experimental conditions as described herein (Supplementary Table S1). For example, the ^Nat^Cu-labeled NOTA-R954 conjugate without the βAla linker retained its ability to bind and effectively antagonize B1R (see peptide MAB7090: IC_50_ values 13 nM and 10 nM, respectively), suggesting that the linker is not essential for its function. Similarly, the ^Nat^Cu-labeled NOTA-R954 conjugate, in which NOTA conjugation occurred at the ε-amino group in the side chain of the extended lysine-containing peptide (Ac-Lys(^Nat^Cu/NOTA)-R954) rather than at the peptide’s N-terminus, also maintained high affinity toward B1R (see peptide MAB8082: IC_50_ value: 19 nM). In parallel, we expanded our analysis by developing new NOTA–agonist peptide conjugates based on our previously developed biostable, high-affinity, and potent B1R agonists for comparative evaluation (Supplementary Table S2) [43,66]. This includes the peptide agonist NG29, which exhibits superior safety and tolerability compared to the antagonist R954 [43,66]. Interestingly, NG29 may also enhance tumor vascular permeability, potentially facilitating better radiopharmaceutical penetration, retention, and deeper tumor infiltration, and ultimately contributing to enhanced radiotherapy efficacy [33,37,67]. Notably, the two related potent Cu-labeled theranostic agonist prototypes, ^Nat^Cu/NOTA-βAla-Lys[Hyp^3^,Igl^5^,dPhe^8^]desArg^9^BK (MAB7095: IC_50_ value: 1.3 nM) and Sar-Lys(^Nat^Cu/NOTA)-Lys[dPhe^8^]desArg^9^BK (MAB8084; IC_50_ value: 2.6 nM), stand out as promising candidates. The theranostic potential of these ^64^Cu-labelled compounds could be further evaluated in future in vivo studies using PET imaging and PRRT to identify the most suitable theranostic candidate for both diagnostic and therapeutic applications against certain types of solid cancer.

In conclusion, our study further emphasized the clinical relevance of B1R in PCa. Here, we have developed the first effective kinin B1R-targeting, R954-based theranostic agent for PCa, featuring favorable pharmacological properties, with the ability to detect and treat prostate tumor lesions in preclinical settings. Further research is needed to determine whether the promising theranostic agent ^64^Cu/NOTA-βAla-R954, along with other candidates described herein, can drive advancements in B1R-positive PCa and other malignancies.

## Figures and Tables

**Table 1:** Binding affinity, selectivity, and potency estimates of a novel NOTA-R954 peptide conjugate at recombinant (HEK293, CHO) and native (HUV) human kinin B1R and B2R.

Sequence	B1R HEK293 IC_50_ (nM)	B2R HEK293 IC_50_ (µM)	B1R-NFAT CHO Kb (nM)	B1R HUV IC_50_ (nM)	B2R HUV IC_50_ (µM)
Ac-Orn-Arg-Oic-Pro-Gly-(αMe)Phe-Ser-DβNal-Ile-OH (R954)	2	>10	1.4	3	>10
NOTA-βAla-Orn-Arg-Oic-Pro-Gly-(αMe)Phe-Ser-DβNal-Ile-OH	11	>10	0.4	9	>10
^Nat^Cu/NOTA-βAla-Orn-Arg-Oic-Pro-Gly-(αMe)Phe-Ser-DβNal-Ile-OH	13	>10	2.2	5	>10

Binding affinities of compounds were expressed in terms of IC_50_ values. Data are means of 2 independent experiments performed in triplicates. For in vitro functional studies (CHO-eGFP/NFAT reporter cells), competitive antagonist affinity estimates (Kb) were obtained from complete agonist concentration–response curves using the Schild simplified Gaddum’s equation: Kb = [B]/(concentration-ratio–1), [B]: antagonist concentration, concentration ratio: EC_50_ of agonist + antagonist/EC_50_ of agonist alone. Data represent means from a single experiment performed in triplicates. For ex vivo experiments (HUV contractile bioassays), data are means of 2 independent experiments.

## Data Availability

All data generated or analysed during this study are included in this published article and its supplementary information files or are available from the corresponding author upon reasonable request.

## Supplementary Materials

The following supporting information can be downloaded at: https://www.mdpi.com/article/10.3390/pharmaceutics17091215/s1, Figure S1: Validation of target specificity of the anti-B1R antibodies, LS-A799 and AS434, in HEK293 cells stably transfected with either mock expression vector (control) or human B1R.; Figure S2: Representative images corresponding to the different immunoscores for prostate epithelial cells and the corresponding normal IgG control staining; Figure S3: HPLC and mass spectrometry analyses of the synthesized peptides R954 and NOTA-βAla-R954; Figure S4: Flow cytometry analysis of B1R expression in PC3 and LNCap cells; Figure S5: Kaplan–Meier survival analysis of the association between hB1R expression and survival probability in lung and kidney cancers; Table S1: Binding affinity and potency estimates of novel NOTA-R954 peptide conjugates in hB1R-stably expressing HEK293 and CHO cells; Table S2: Binding affinity and potency estimates of novel NOTA–agonist peptide conjugates in hB1R-stably expressing HEK293 and CHO cells.

## Abbreviations

The following abbreviations are used in this manuscript:

PCa	Prostate cancer
FDG	^18^F-fluorodeoxyglucose
PSA	Prostate specific antigen
DRE	Digital rectal examination
MRI	Magnetic resonance imaging
GPCR	G protein-coupled receptors
PET	Positron emission tomography
TMA	Tissue microarray
IHC	Immunohistochemistry
FFPE	Formalin-fixed paraffin-embedded
DMEM	Dulbecco’s modified Eagle medium
FBS	Fetal bovine serum
PIN	Prostatic intraepithelial neoplasia
