# Supplementary material for: Theranostic Potential of a New 64Cu-Labeled NOTA-R954 Peptide Conjugate for Kinin B1R Expressing Prostate Cancer
Source: Pharmaceutics. 2025 Sep 18;17(9):1215. doi: 10.3390/pharmaceutics17091215 (PMC12473165; doi:10.3390/pharmaceutics17091215)
Supplement: Supplementary file 1 [file pharmaceutics-17-01215-s001.zip › pharmaceutics-3829769-supplementary.pdf]

Supplementary Material

Theranostic Potential of a New ^{64}Cu-Labeled NOTA-R954 Peptide Conjugate for Kinin B1R Expressing Prostate Cancer

Sadaf Ghanaatgar Kasbi [1,2], Martin Savard [1,2], Frédéric Couture [3], Céléna Dubuc [1,2], Véronique Dumulon-Perreault [4], Marie-Edith Nepveu-Traversy [1,2], Samia Ait-Mohand [4], Robert Sabbagh [5], Sameh Geha [6], Brigitte Guérin [2,4], Yves Dory [7] and Fernand Gobeil [1,2,*]

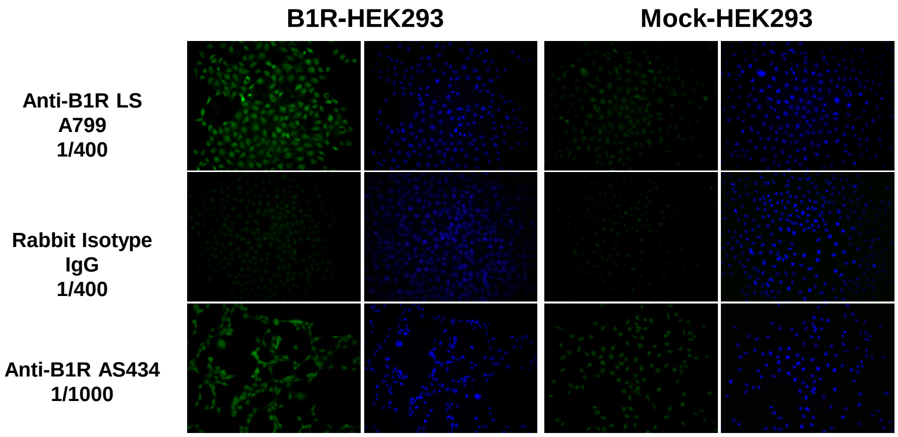

Figure S1. Validation of target specificity of the anti-hB1R antibodies, LS-A799 and AS434, in HEK293 cells stably transfected with either mock expression vector (control) or hB1R. Validation of antibody specificity was assessed by fluorescence immunocytochemistry on pre-blocked, paraformaldehyde-fixed/saponin-permeabilized cells. Magnification: 20x. Primary antibodies diluted as indicated in PBS pH 7.4/0.1% saponin/5% goat serum/5% horse serum. Secondary antibody: Alexa Fluor 488 anti-rabbit secondary antibody diluted (1/300) in PBS pH 7.4/0.1% saponin/5% goat serum/5% horse serum. Counterstaining with Hoechst 33342 (100 nM). All sets of images were digitally adjusted in terms of brightness/contrast/gamma values under the same parameters using Adobe Photoshop (version CS6; Adobe Inc., San Jose, CA, USA). Note the relatively higher staining intensities obtained with the two primary antibodies in hB1R transfectants compared to mock cells as assessed by epifluorescence microscopy (Leica DM4000; Leica Microsystems, Concord, ON, Canada). Negative controls with an isotype-matched rabbit IgG (diluted 1/400) showed almost no staining. Representative photomicrographs from two independent experiments.

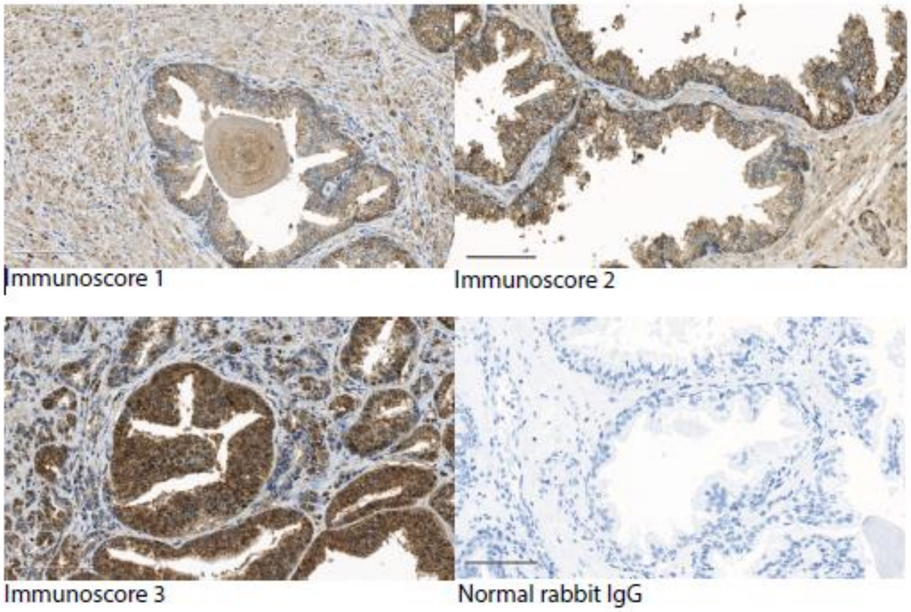

Figure S2. Representative images corresponding to the different immunoscores for prostate epithelial cells and the corresponding normal IgG control staining.

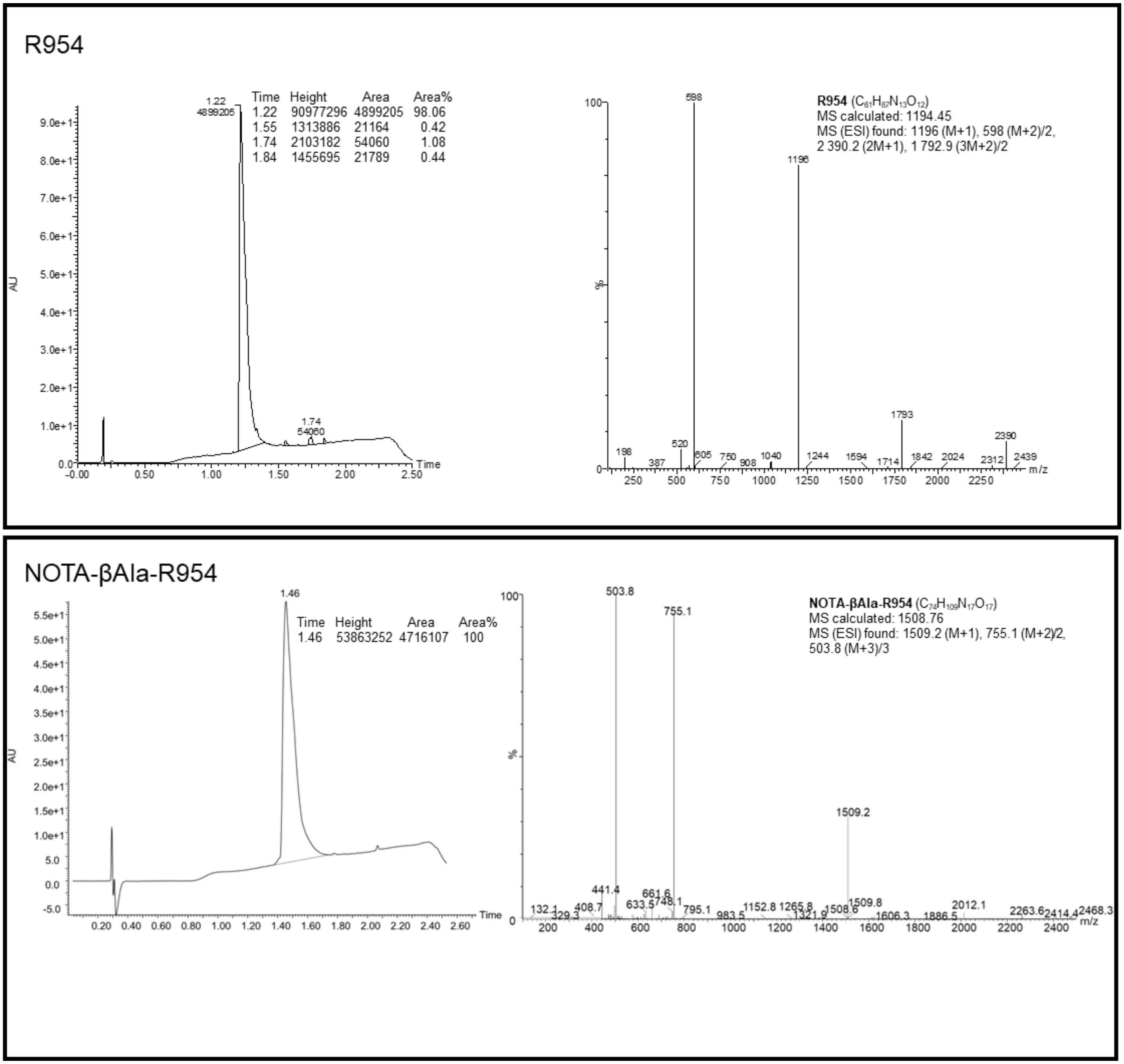

Figure S3. Representative HPLC chromatogram and corresponding mass spectra of the synthesized B1R peptide antagonists: R954 (upper panel) and NOTA-βAla-R954 (lower panel). Reverse-phase HPLC chromatogram showed a dominant peak at 1.22 min (98.06% area) for R954 and at 1.46 min (100% area) for NOTA-βAla-R954. In both cases, the mass spectrum of the major peak displayed the expected molecular/protonated ion(s), confirming the identity of the target compound (MS calculated vs. MS (ESI) found). The corresponding *m*/*z* values are shown on each spectrum. Data are representative of *n*= 1–3 independent runs.

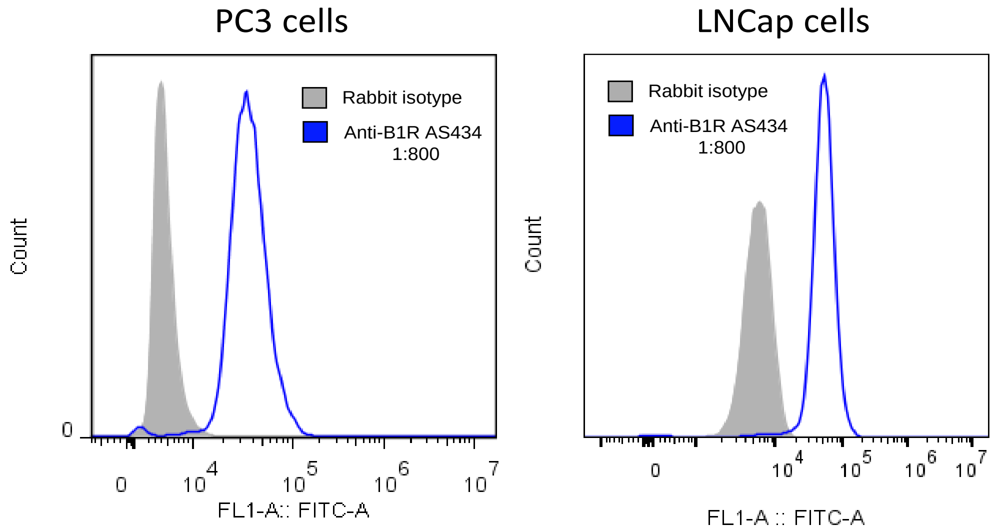

Figure S4. Flow cytometry analysis of B1R expression in PC3 and LNCap cells. PC3 and LNCap cells were washed three times in PBS supplemented with 0.5% BSA (fluorescence-activated cell sorting buffer) in the presence of Fc receptor blocking reagent (1.25 µg/10^6 cells, BD Bioscience, San Jose, CA) and stained with isotype nonimmune rabbit IgG (1.25 µg/mL) or anti-hB1R antisera AS434 (1.25 µg/ml) per 10^6 cells for 30 min at room temperature. Cells were washed with 1.0 mL fluorescence-activated cell sorting buffer to remove unbound antibodies. After the final wash, labeled cells were fixed in 500 µL of 2% paraformaldehyde in PBS and 10^5 cells were analyzed by flow cytometry using a FACScan flow cytometer and CellQuest software (BD Pharmingen, San Diego, CA, USA).

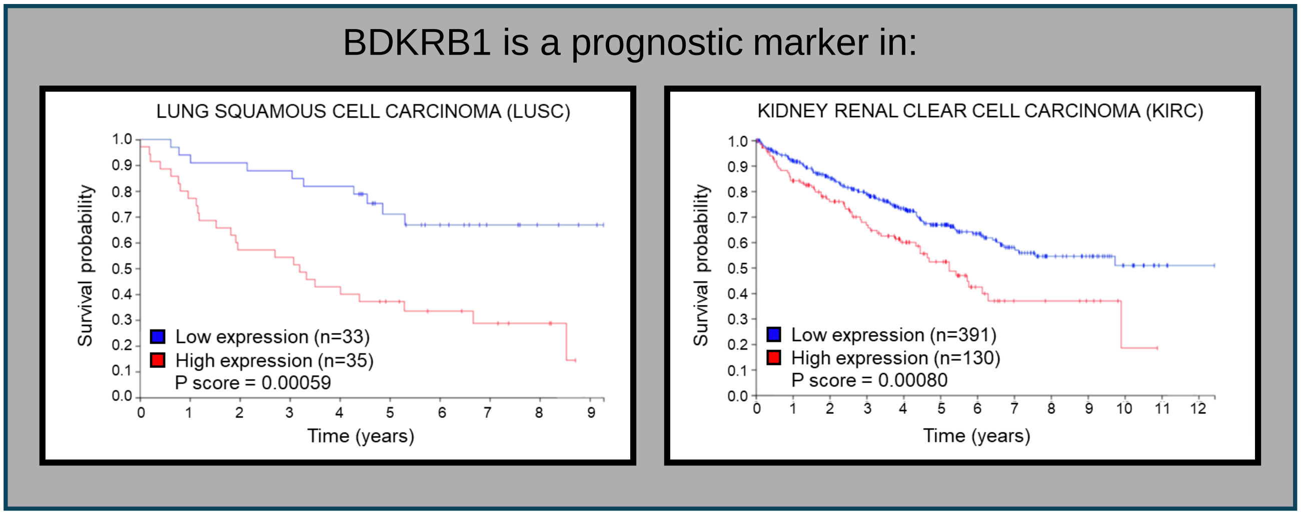

Figure S5. Kaplan–Meier survival analysis of the association between hB1R expression and survival probability in LUSC and KIRC cohorts. The size of each patient group and the log-rank p-value are reported. Modified from: https://www.proteinatlas.org/ENSG00000100739-BDKRB1/cancer/renal+cancer#KIRC_validation.

Table S1. Binding affinity and potency estimates of novel NOTA-R954 peptide conjugates in hB1R-stably expressing HEK293 and CHO cells

Codename	Sequence	HEK293 IC$_{50}$ (nM)	CHO-NFAT IC$_{50}$ (nM)
R954	Ac-Orn-Arg-Oic-Pro-Gly-(αMe)Phe-Ser-DβNal-Ile-OH	2	1.4
MAB7019	NOTA-βAla-Orn-Arg-Oic-Pro-Gly-(αMe)Phe-Ser-DβNal-Ile-OH	11	0.4
MAB7049	^{Nat}Cu/NOTA-βAla-Orn-Arg-Oic-Pro-Gly-(αMe)Phe-Ser-DβNal-Ile-OH	13	2.2
MAB7020	NOTA-Orn-Arg-Oic-Pro-Gly-(αMe)Phe-Ser-DβNal-Ile-OH	5	0.4
MAB7090	^{Nat}Cu/NOTA-Orn-Arg-Oic-Pro-Gly-(αMe)Phe-Ser-DβNal-Ile-OH	13	9.8
MAB8065	Ac-Lys(NOTA)-Orn-Arg-Oic-Pro-Gly-αMePhe-Ser-DβNal-Ile-OH	12	n.d.
MAB8082	Ac-Lys(^{Nat}Cu/NOTA)-Orn-Arg-Oic-Pro-Gly-αMePhe-Ser-DβNal-Ile-OH	19	n.d.

Data are means of two independent experiments. Abbreviations: n.d.: not determined. The purity and identity of the synthesized peptides were confirmed using ultra-performance liquid chromatography coupled with tandem mass spectrometry (UPLC-UV-MS, Waters AQUITY-H-Class-SQD2, column Waters BEH C18 (1.7 μm, 2.1 x 50 mm); Waters Corporation, Milford, MA, USA). Note that for the peptides MAB8065 and MAB8082, NOTA chelators were conjugated not to the N-terminus of the peptide but to the ε-amino group in the side chain of extended lysine-containing peptides. This alternative conjugation site appears to be well tolerated, with no observable impact on B1R affinity.

Table S2. Binding affinity and potency estimates of novel NOTA–agonist peptide conjugates in hB1R-stably expressing HEK293 and CHO cells

Codename	Sequence	HEK293 IC$_{50}$ (nM)	CHO-NFAT EC$_{50}$ (nM)
DBK *	Arg-Pro-Pro-Gly-Phe-Ser-Pro-Phe-OH (DBK)*	102	297
LDBK *	Lys-Arg-Pro-Pro-Gly-Phe-Ser-Pro-Phe-OH (LDBK)*	0.9	2.1
MAB2047	NOTA-βAla-Lys-Arg-Pro-Pro-Gly-Phe-Ser-Pro-DPhe-OH	2.3	2.2
MAB7088	NOTA-βAla-Lys-Arg-Pro-Hyp-Gly-Igl-Ser-Pro-DPhe-OH	0.3	0.1
MAB7095	^{Nat}Cu/NOTA-βAla-Lys-Arg-Pro-Hyp-Gly-Igl-Ser-Pro-DPhe-OH	1.3	n.d.
MAB8066	Sar-Lys(NOTA)-Arg-Pro-Pro-Gly-Phe-Ser-Pro-DPhe-OH	40.0	41.0
MAB8073	Sar-Lys(NOTA)-Lys-Arg-Pro-Pro-Gly-Phe-Ser-Pro-DPhe-OH	1.1	1.0
MAB8084	Sar-Lys(^{Nat}Cu/NOTA)-Lys-Arg-Pro-Pro-Gly-Phe-Ser-Pro-DPhe-OH	2.6	1.3

* Selective natural agonists for the hB1R. Data are means of two independent experiments. n.d.: not determined. As with some B1R antagonist conjugates, NOTA was conjugated to the ε-amino group of the lysine side chain in the extended lysine-containing B1R agonist peptides. This modification was shown to be permissive, with no detectable impact on receptor affinity or biological activity (see peptides MAB8073 and MAB8084). Abbreviations for amino acids are described as follows: Hyp, trans-4-hydroxy-L-proline; Igl, 2-indanyl-glycine; Sar, N-methyl-glycine.
